# Rigg's Disease, Pyorrhea Alveolaris, or So-Called Interstitial Gingivitis, in a Nut Shell

**Published:** 1904-08

**Authors:** B. F. Arrington

**Affiliations:** Goldsboro, N. C.


### RIGG'S DISEASE, PYORRHEA ALVEOLARIS, OR SO-CALLED INTERSTITIAL GINGIVITIS, IN A NUT SHELL.

By B. F. Arrington, D. D. S.,
Goldsboro, N. C.

(Written for *The American Journal of Dental Science.*)

This disease call it what we may matters not, so we diagnose correctly and treat successfully. Scurvy is the name by which it has been longest known, and is most generally spoken of by the masses. Gingiva pericementitis or periastitis would possibly be more expressive and correct, locating point of first perception and defining limit of disease.

It is unquestionably a local disease of small compass, and not constitutional or partly so, as some contend, as evidenced in failure to check and cure by constitutional means after long months and years of treatment by some of the ablest men in the profession. Whereas by local treatment it is easily checked and cured.

The disease is confined exclusively, except as to features of sequence, to the membrane lining walls of teeth sockets and covering roots of teeth from apex to alveolar margin. The first symptom of the disease always presents unmistakably, at gum margin, and seldom before the age of ten, and rarely after forty or forty-five.

The cause, not definitely agreed upon, unquestionably is local, and may consist, chiefly if not exclusively in neglect of removal of impacted food substances around the necks of teeth until putrefaction and poison affecting the membrane above named the result, with serious consequences following, destruction of membrane, waste of process and loss of teeth. To prevent this unfortunate state of disease and loss of membrane, alveolus and teeth, the daily use of tooth brush with plenty of water in mouth after meals, for cleansing teeth and removing debris from mouth, as a life practice, commencing with the first eruption of permanent teeth there would be but seldom if ever a trace of the disease. Likewise, after treatment and cure is effected, if brush and water is systematically used as good judgment should dictate, there would be no return of the disease. These assertions are based on facts realized in practice, treatment recorded and results watched.

To thoroughly remove the deposits from roots of teeth, guarding against injury to cementum is first feature and first duty in treatment, then the free use of dilute sulphuric acid and pulverized pumice stone for perfect cleansing of teeth, and wholesome effect of the acid upon the soft tissues and wasting alveolus, followed by a liberal application of campho-phenique or other suitable remedy for the healing of soft tissues, as the winding up finish in treatment is all that is required for check and cure of the disease, and one treatment at one sitting is quite sufficient in the majority of cases. But few cases, the most extreme, with loosened teeth and free discharge of pus do not require more than from four to six weeks to effect a cure, and no special need for professional attention after two or three days following removal of deposits. The teeth will not tighten in sockets so soon, but that feature of restoration to a normal status, is only a question of limited time if instructions in use of brush and water are persistently followed.

I will venture to say without fear of truthful contradiction that a large majority of all the varied phases of the disease so extravagantly mentioned by some writers, claimed to be produced by sundry *local* and *constitutional causes* (very absurd) can be successfully treated by the simple line of treatment above mentioned. I am sure the fact would be definitely realized in every case where there was positive evidence of deposits on roots of teeth for such and none other are genuine cases of the disease.

For more than twenty years this disease has been extravagantly written about and discussed, most sensational and impractical and void of good results. Some of the ablest men in the profession, men that have done much for the profession on various lines have sidetracked wonderfully and blunderingly, and displayed great weakness in discussing this subject, evidencing much ignorance pertaining to the disease, and demonstrating easily the fact to all reasoning unprejudiced minds, that they weremore sensational and speculative in theory, than conservative and practical in practice. When a dentist (matters not who he is or how prominent) speaks of this disease as the *effect* of *numerous constitutional* and *local causes,* and advises in treatment the use of a multiplicity of remedies, and go to the extent that some do, in advising use of compressed air with force from fifty to seventy-five pounds, cataphoresis, cutting and scraping away the cementum and alveolar process, devitalizing nerve pulps, cutting off crowns of teeth and crowning, the application of lactic, tricheorecetic, sulphuric and other acids for softening and removal of deposits, and prescribe blood letting, Turkish baths, a dose of epsom salts and other remedies every day for weeks, general constitutional treatment, pointing remedies to kidneys for check and cure of the disease, you may mark it down in plain letters, that they are ignorant on the subject, and are indulging much guess work in theorizing and practice, and have not the slightest practical conception of the true character of the disease, therefore are not safe guides to follow after in practice. In my judgment, their much writing and talking on such unreasonable lines of procedure in treatment, has done more harm than good to the profession and to those they have treated.

What we want and most need in treatment and for cure of the disease, is a fair amount of plain, practical common-sense reasoning, with practical conformity to nature's requirements and less of the speculative, sensational, theorizing guess work that has proven so confusing and misleading. This fact is sustained by daily observation of results that follow treatment. Reliable, safe practice must and will come to the front. It is being demonstrated in daily practice, and all that is requisite for acceptance of the truth, is to stand by and witness practical demonstrations, and to record and watch results.

There may be some doubting Thomases in the ranks who will not willingly accept plain truth statements established through practice, unless considerably tinged with the sensational, such is the measure and make-up of some dentist, a weak feature and to be regretted.

By waiting and working faithfully on conservative lines, we will realize with great satisfaction that what we call Rigg's disease, though a very prevalent and troublesome disease (mostly for want of a correct comprehension of cause) is not a very big thing after all, and is subject to cure and permanent relief by moderate treatment, and the control of every dentist who is willing to venture experimental practice to determine if statements are facts sustainable in practice.

Many dentists when success is realized and there is no further doubt on mind, will possibly often smile when thinking of the many unreasonable, amusing and absurd theories and practices proclaimed by prominent dentists, pertaining to cause and treatment of this very common and easily treated disease.

I will suggest to reflect and let reason rule for a right conception of cause, and moderate, successful treatment of what is known as Rigg's disease.

The disease is almost as common with certain classes as tooth decay, and dentists generally can, if they will, after a little experience, treat it easily and as successfully as failing teeth. Then all this sensational talk and writing on the subject, and the much unreasonable, out of line treatment will cease, and by the lapse of a decade or two, extreme typical cases will but seldom present for treatment.

The distinguished Dr. James Truman of Philadelphia, of more than fifty years' experience in dental practice, and for many years a conspicuous educator as professor in one of the leading dental colleges of our country, in speaking of this disease, has said: "Eternal vigilance is the price we must all pay for health." A truth indisputable, therefore daily prophylactic treatment should be freely indulged for the perpetuation of a healthy state of teeth and gums. This done, much will be accomplished, all will come right in a reasonable period of time, and but little will be said or heard of Rigg's disease, from the fact, that dentists generally will be treating it as successfully as any other service in dental practice.

---

### COHESIVE TIN.

By W. M. Randall, D. D. S.

Professor of Operative Technics, Dental Anatomy and Comparative Dental Anatomy in the Louisville College of Dentistry.

(Written for *The American Journal of Dental Science.*)

